# Influenza A(H11N2) Virus Detection in Fecal Samples from Adélie (*Pygoscelis adeliae*) and Chinstrap (*Pygoscelis antarcticus*) Penguins, Penguin Island, Antarctica

**DOI:** 10.1128/spectrum.01427-22

**Published:** 2022-09-19

**Authors:** Maria Ogrzewalska, Fernando Couto Motta, Paola Cristina Resende, Tulio Fumian, Ana Carolina Fonseca da Mendonça, Luciana Appolinario Reis, Martha Lima Brandao, Marcia Chame, Ighor Leonardo Arantes Gomes, Marilda Mendonca Siqueira

**Affiliations:** a Laboratory of Respiratory Viruses and Measles, Oswaldo Cruz Foundation, FIOCRUZ, Rio de Janeiro, Brazil; b Laboratory of Comparative and Environmental Virology, Oswaldo Cruz Institute, FIOCRUZ, Rio de Janeiro, Brazil; c Institutional Platform for Biodiversity and Wildlife Health, FIOCRUZ, Rio de Janeiro, Brazil; University of Georgia

**Keywords:** avian influenza, H11N2, virus, Antarctica, penguin

## Abstract

Influenza A viruses infect a range of host species, including a large variety of mammals and more than a hundred species of birds. A total of 95 avian fecal samples were collected from penguin colonies in the South Shetland Islands, close to the Antarctic Peninsula, and tested by reverse transcription-PCR (RT-PCR) to detect avian influenza viruses (AIVs). Five out of seven samples collected from Penguin Island were positive for AIVs. Analysis of the genomes recovered from four samples revealed the detection of influenza A(H11N2) virus in fecal samples from Adélie penguins (Pygoscelis adeliae) and from a colony of chinstrap penguins (Pygoscelis antarcticus). Bayesian phylogeographic analysis revealed the clustering of all currently available H11N2 samples from Antarctica’s avifauna in a single cluster that emerged at least in the early 2010s, suggesting its continued circulation on the continent. Our results reinforce the need for continuous surveillance of avian influenza on the Antarctic continent.

**IMPORTANCE** Although wild birds play a role in the transmission and ecology of avian influenza viruses (AIVs) across the globe, there are significant gaps in our understanding of the worldwide distribution of these viruses in polar environments. In this study, using molecular analysis and full-genome sequencing, we describe the detection of distinct influenza A(H11N2) viruses in fecal samples of penguins in the Southern Shetland Islands, Antarctica. We emphasize the need for virus monitoring as AIVs may have implications for the health of endemic fauna and the potential risk of the introduction of highly pathogenic AIVs to the continent.

## INTRODUCTION

Influenza A viruses (genus *Alphainfluenzavirus*), members of the *Orthomyxoviridae* family, are classified into subtypes based on the antigenic properties of the two surface glycoproteins hemagglutinin (HA) and neuraminidase (NA). Currently, 18 HA (H1 to H18) and 11 NA (N1 to N11) subtypes have been described, including H17N10 and H18N11, detected in fruit bats in Central and South America (1, 2). Influenza viruses have been detected in terrestrial, flying, and aquatic mammals as well as in a large number of different wild aquatic birds such as Anseriformes (teals, ducks, geese, and swans) and Charadriiformes (shorebirds, gulls, and terns), which are considered the natural reservoirs of avian influenza viruses (AIVs) (1–6).

The regular long-distance migratory behavior of these birds has a direct impact on the spread of AIVs across countries or even continents (7). Birds breeding in one geographic region regularly follow similar migratory flyways, allowing the connection of many bird populations in time and space, either at common breeding areas, during migration, or in shared nonbreeding areas. Consequently, virus-infected birds may transmit their pathogens to other populations, which can then bring the viruses to new areas (7).

Antarctica is the most southern and isolated continent on earth; however, many research groups have been able to investigate AIVs in its native avifauna. Previous studies have reported AIV antibody-positive serum samples obtained from Adélie (Pygoscelis adeliae), chinstrap (Pygoscelis antarcticus), and gentoo (Pygoscelis papua) penguins; south polar skuas (Stercorarius maccormicki); and southern giant petrels (Macronectes giganteus) (8–13). This serological evidence demonstrates that AIV infection is widespread and prevalent in Antarctic birds; however, the pathogenic and genetic characterization of AIVs in these populations remains limited. Genetic information on AIVs from the Antarctic continent was first described only in 2014, when an AIV subtype H11N2 strain was identified in P. adeliae from a breeding colony on the Antarctic Peninsula (14). Later, in 2015, the same AIV H11 strain was detected in a snowy sheathbill (Chionis albus), and AIV subtype H5N5 strains were detected in chinstrap penguins (P. antarcticus) (15, 16). Phylogenetic analysis of the HA gene showed its relationship with H5 low-pathogenicity North American lineage viruses, and analysis of the NA gene showed its relationship with N5 viruses from Eurasia (16). More recently, the detection of an influenza A(H4N7) virus in M. giganteus (17) added more information about the ecology of AIVs in Antarctica; however, knowledge about the influenza virus distribution and the circulation of distinct subtypes in Antarctica fauna remains extremely limited.

H11N2 subtype AIVs frequently infect wild and domestic birds across the world (18–23); so far, H11 viruses have not been detected in humans, and together with its limited ability to grow in mammals (20), it is likely that this subtype has a low zoonotic potential for mammals. However, one strain of H11N6 has been isolated from swine in South Korea (A/swine/KU/2/2001 H11N6 [EPI_ISL_80217]), and the exposure of sea otters has been serologically confirmed (24), which may indicate that H11 influenza viruses possess the ability to cross the species barrier to infect mammals.

In the present study, we report influenza A subtype H11N2 viruses detected in fresh fecal samples from penguins in the Southern Shetland Islands, Antarctica, adding new data about the natural history and ecology of AIVs in Antarctica.

## RESULTS AND DISCUSSION

**FIG 1:**
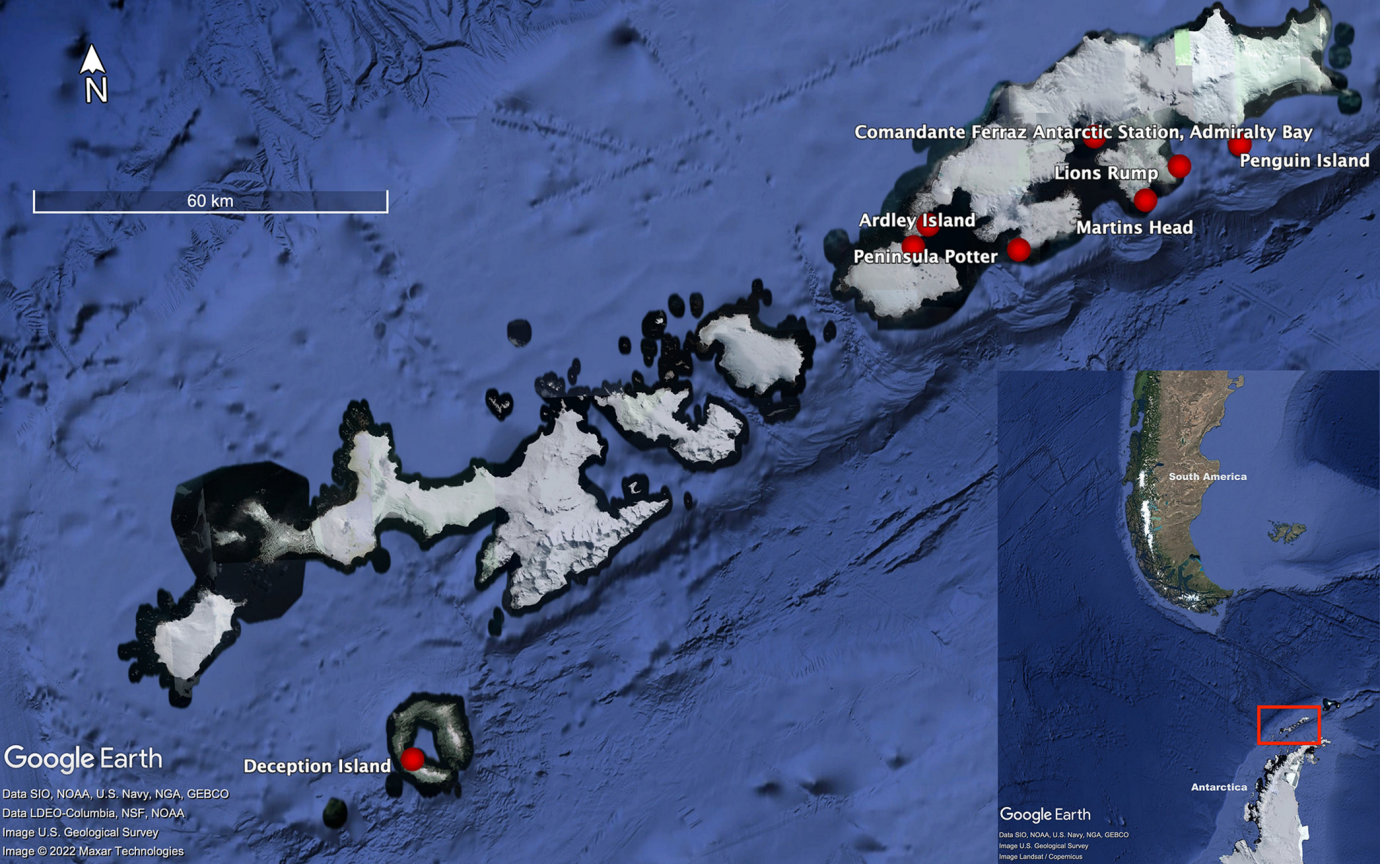
Localization of the South Shetland Islands (small image) and collection sites (red dots) in the present study, November 2019 to January 2020 (large image).

Various research studies have shown that Antarctic avifauna is infected or acts as a host for several avian microorganisms, including AIVs (9, 25–28). In the present study, a total of 95 avian fecal samples collected from eight localities (Comandante Ferraz Antarctic Station [27 samples], Potter Peninsula [16], Lions Rump [11], Martins Head [8], Rip Point [3], Ardley Island [17], Penguin Island [7], and Deception Island [6]) were tested by reverse transcription-PCR (RT-PCR) targeting the M gene to detect AIVs (Fig. 1). Five out of seven samples collected from Penguin Island were positive (threshold cycle [*C_T_*] values ranging from 25.9 to 39.2); four of these samples were collected from the environment of the colony of *P. antarcticus* penguins, and one sample was collected immediately after defecation from one isolated *P. adeliae* penguin (Marcia Chame, unpublished observation). All of these samples were collected on the same day, but the distance between the collected samples was always >5 m. AIV whole genomes were recovered from samples F168/2020 and F171/2021, and partial genomes were recovered from samples F162/2020 and F163/2020. Despite AIV positivity by RT-PCR (*C_T_* = 39.2), no gene sequence was recovered from sample F167/2020. The acquired genome sequences were uploaded to the EpiFlu database of the GISAID (Global Initiative on Sharing Avian Influenza Data) (www.gisaid.org) (Table 1).

**TABLE 1:** Real-time RT-PCR-positive environmental fecal samples from Penguin Island and the South Shetland Islands, Antarctica, January 2020

Sample	Predominant bird species in the colony^*a*^	*C_T_* value^*b*^	Genome segment(s) detected^*c*^	Virus name	GISAID accession no.
F162/2020	*Pygoscelis antarcticus*	36.1	PA, NA, MP, NS	A/environmental/Antarctica/F162/2020	EPI_ISL_2397324
F163/2020	*Pygoscelis antarcticus*	37.3	MP	A/environmental/Antarctica/F163/2020	EPI_ISL_2397326
F167/2020	*Pygoscelis antarcticus*	39.2	None		
F168/2020	*Pygoscelis antarcticus*	25.9	Complete	A/environmental/Antarctica/F168/2020	EPI_ISL_2397327
F171/2020	*Pygoscelis adeliae*	38.9	Complete	A/environmental/Antarctica/F171/2020	EPI_ISL_2397515

a All of these samples, with the exception of one from a *Pygoscelis adeliae* individual found outside the colony, were collected from colonies of *Pygoscelis antarctica* penguins. However, the presence of other bird species such as skuas, petrels, and snowy sheathbills was always observed in these colonies.

b *C_T_*, threshold cycle by real-time RT-PCR targeting the matrix (M) gene.

c Segments of the influenza virus genome sequenced. PA, RNA polymerase subunit; NA, neuraminidase; MP, matrix protein; NS, nonstructural protein. Complete indicates that all eight segments were sequenced.

This is the first detection of influenza A(H11N2) virus on Penguin Island. However, the same subtype, H11N2, was previously detected in a colony of *P. adeliae* from Admiralty Bay, King Gorge Island (2013); in C. albus from Kopaitik Island, Antarctic Peninsula (2014); and in *P. antarcticus* from Cape Shirreff, Livingston Island (2017), approximately 30, 135, and 150 km away from Penguin Island, respectively (14, 15), suggesting that H11N2 continues to circulate among wild birds in the region of the Antarctic Peninsula and South Shetlands Islands and probably even further, as penguin species have migratory behavior during the winter months, spreading over hundreds of kilometers from their breeding colonies (29). For example, chinstrap penguins migrate by swimming up to 4,500 km away from their colonies to overwinter in a latitude near 60°S in the Southern Ocean; those from the western Antarctic Peninsula travel mostly westward (30, 31). Adélie penguins from the northern Antarctic Peninsula migrate east to overwinter in the Weddell Sea pack ice zone, remaining away from their colonies for approximately 9 months, at distances of up to 2,000 km (31, 32).

**FIG 2:**
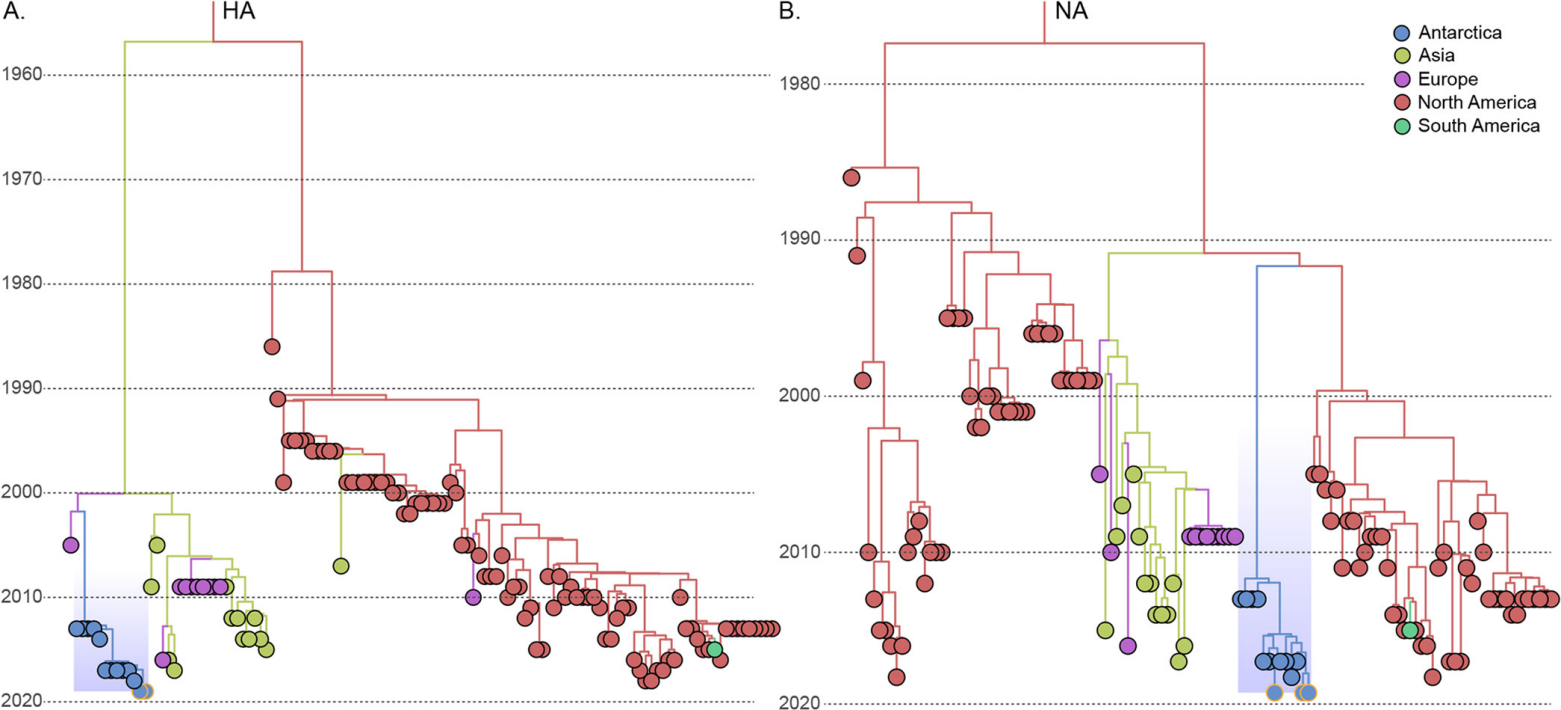
Phylogeographic analysis of the hemagglutinin and neuraminidase genes of the influenza A virus H11N2 samples from Antarctica. The time-scaled Bayesian maximum clade credibility (MCC) trees of the HA (*n *= 123) (A) and NA (*n *= 125) (B) genes are shown. The inherent locations of tips and nodes in the trees are represented according to the color key in the top right corner. The Antarctic cluster is highlighted in light blue in both trees. Sequences from this study are highlighted by orange outlines.

Our phylogeographic analysis revealed, in all eight gene segments, the clustering of all available Antarctic H11N2 AIVs, whether from *P. adeliae* or *P. antarcticus* (14, 15), in a highly supported cluster (posterior probability of 1.0 for all eight gene segments) (Fig. 2A and B; see also Fig. S2A to F in the supplemental material). The inspection of the temporal structure revealed a significant association (*P* < 0.5) between elapsed time and molecular divergence in seven of the eight H11N2 gene segments (Fig. S1). Indeed, across the eight gene segments analyzed, the median molecular clock values mostly converged to values within previously estimated intervals (Table S2) (15, 33). The time of the most recent common ancestor (*T*_MRCA_) inferred for the Antarctic cluster in this analysis (Table S3) suggested the circulation of the H11N2 cluster in the continent at least since the late 2000s. In all trees but the HA one, the detected cluster had a common ancestor outside Antarctica in North America, with various posterior state probabilities (PSPs) (1.00 to 0.35). Nonetheless, the HA cluster was more closely associated with European diversity, with a significant PSP (0.61). Nonetheless, the small representation of South American (0.4%) and Antarctic (0.002%) samples in the EpiFlu AIV H11N2 data set can certainly cloud both potential dissemination routes and the real diversity within Antarctica. Among the inferred topologies, (i) the clustering of all available sequences, (ii) the long branch connecting the Antarctic H11N2 cluster to its first common ancestors outside the continent (mean of ~35 years), and (iii) the geographic history shared by the majority of the gene segments of H11N2 are all arguments that validate the previously postulated hypothesis of the compartmentalized evolution of the H11N2 subtype in Antarctica (14, 15).

It was hypothesized previously that new strains of AIVs are introduced into the Antarctic ecosystem only on rare occasions (14). Once introduced, however, these viruses may become endemic within the local bird population and over time become highly divergent from other AIVs on the planet. Our results support this proposal. However, other studies demonstrated the more recent introduction of a novel AIV H5N5 reassortant, showing that the introduction of new AIVs to Antarctica may be more frequent than previously thought (15, 16). In fact, each spring, over 100 million birds (mainly penguins but also skuas, sheathbills, gulls, petrels, sterns, and shags) breed around the rocky Antarctic coastline and offshore islands. They gather in huge and dense colonies, sharing habitats and enabling close contact. During the winter many species of these birds, such as Antarctic terns (Sterna vittata) or *Macronectes* spp., visit the coast or coastal waters of South America, Africa, Australia, or New Zealand and/or inhabited Subantarctic islands (17, 29), other species breed in Antarctica and overwinter in the Northern Hemisphere, such as south polar skuas (S. maccormicki) (34), or breed in other regions and overwinter in Antarctica, such as Arctic terns (Sterna paradisaea) (35). Such wildlife migration demonstrates the potential route of transmission of AIVs, with risks of migratory species encountering the virus in regions on their migration paths and introducing these viruses to penguin colonies. Thus, especially sterns, gulls, skuas, and petrels might represent natural hosts that play a role in the introduction and maintenance of AIVs in Antarctica (17, 36).

We are aware of the limitations of our study. The fact that we did not find influenza virus in other areas does not exclude the possibility of its presence, as direct detection by RT-PCR screening of viral infections underestimates prevalence (36). The prevalence of influenza in penguin species is generally low as determined by RT-PCR, less than 5% (14–16), in contrast to prevalence evaluated by serological screening reaching 8.8 to 12.0% and 7.4 to 26.9% in *P. adeliae* and *P*. *antarcticus*, respectively, and even 42% in macaroni penguins (Eudyptes chrysolophus) (11, 12, 14, 36). A similar situation is observed for other viruses; for example, some avian avulaviruses (avian orthoavulaviruses 1, 10, 17, 18, and 19) were described to be present at low rates of occurrence of 6% in P. papua penguins (37) and 7% in *P. adeliae* penguins (38). However, when serology was performed, higher levels of seroprevalence were determined: 30.3% in Magellanic penguins (Spheniscus magellanicus) (39) and 37% in *P. antarcticus* penguins (40); thus, both molecular and serological methods are needed to understand AIVs in Antarctica.

Although it is well established that wild-bird species play a role in the transmission and ecology of AIVs, there are significant gaps in our understanding of the worldwide distribution of these viruses, specifically the prevalence, geographic distribution, and/or significance of AIVs in polar environments. To increase our understanding of the complex relationship of AIVs with their hosts in these environments, it is crucial to integrate virus and host ecology within long-term surveillance studies. Our findings reinforce the persistence of divergent H11N2 viruses among Antarctic bird wildlife and emphasize the monitoring of the occurrence of viruses in samples of excreta in bird habitats using molecular detection methods and noninvasive collection methods.

## MATERIALS AND METHODS

### Ethical aspects.

Permission to collect samples was granted by the Environmental Assessment Group of the Brazilian Antarctic Program (GAAm-PROANTAR XXXVIII) at Potter Peninsula (ASPA 132); Lions Rump (ASPA 151); Martins Head, Ardley Island (ASPA 150); and Deception Island (ASPA140). Penguin Island is an important bird area (IBA) with no access restrictions.

### Sample collection.

Fecal samples were collected throughout November and January to February of the 2019–2020 breeding season in the Antarctic summer season during two field expeditions carried out in the South Shetland Islands close to the Antarctic Peninsula. Sampling occurred in eight localities in total. On King George Island, samples were collected close to the Comandante Ferraz Antarctic Station in Admiralty Bay (62°5′S, 58°23′W), Potter Peninsula (62°15′S, 58°38′W), Lions Rump (58°08′W, 62°08′S), and Martins Head (62°11′S, 58°14′W). On Nelson Island, samples were collected from Rip Point (62°14′S, 58°58′W), Ardley Island (62°12′S, 58°55′W), Penguin Island (62°6′S, 57°55′W), and Deception Island (62°58′S, 60°41′W) (Fig. 1).

Both individual fresh samples from monitored animals and pools of feces from the penguins’ nesting sites were collected. Fecal material was collected with sterile Dacron swabs, which were immediately placed into tubes containing 2 mL of viral transport medium composed of Dulbecco’s modified Eagle’s medium (DMEM) cell culture medium supplemented with fetal bovine serum (10%) and antibiotics and antifungals (penicillin at 100 IU/mL, streptomycin at 50 μg/mL, amphotericin B at 0.1 μL/mL, gentamicin at 1,000 μg/mL, and kanamycin sulfate at 650 μg/mL) and kept refrigerated for up to 4 h before being frozen at −80°C.

### Viral RNA extraction.

Clarified fecal suspensions (20%, wt/vol) were prepared with 1× phosphate-buffered saline (PBS) by vortex mixing, followed by centrifugation at 3,000 × *g* for 20 min, and 140 μL of the supernatant was used for viral RNA extraction. Viral RNA was extracted using a QIAamp viral RNA minikit (Qiagen, CA, USA) and a QIAcube automated system (Qiagen), according to the manufacturer’s instructions. Viral RNA was eluted in 60 μL of the elution buffer. The isolated RNA was immediately stored at −80°C until molecular analysis. For each extraction procedure, RNase/DNase-free water was used as a negative control.

### Influenza virus detection and characterization.

Influenza A virus was detected using TaqMan-based quantitative one-step real-time RT-PCR. All reactions were performed using the SuperScript III Platinum one-step quantitative RT-PCR (qRT-PCR) kit (Thermo Fisher Scientific, Invitrogen Division, Carlsbad, CA, USA) according to the protocol established by the Collaborative Influenza Center, Centers for Disease Control and Prevention, Atlanta, GA (41). Samples that crossed the threshold line below a threshold cycle (*C_T_*) value of 40 and showed a characteristic sigmoid curve were regarded as positive.

### Whole-genome sequencing.

For influenza A virus whole-genome sequencing, 8 μL of the viral RNA was used for multisegmented reverse transcription-PCR (M-RT-PCR) using influenza A virus universal primers for the amplification of the eight gene segments of all influenza virus subtypes in a multiplex reaction (42). The generated amplicons were purified using the ExoSAP-IT PCR product cleanup reagent (Invitrogen) and quantified using a Qubit dsDNA (double-stranded DNA) HS assay kit (Thermo Fisher Scientific) according to the manufacturer’s protocols. The cDNA library was constructed using the Nextera XT DNA library preparation kit (Illumina) and submitted to sequencing with the Illumina MiSeq system (Laboratory of Respiratory Viruses and Measles, Oswaldo Cruz Foundation, FIOCRUZ, Rio de Janeiro, Brazil) using MiSeq reagent kit v2 micro (300 cycles; Illumina) (43).

### Bioinformatic pipeline.

The bioinformatic pipeline to assemble the reads and obtain the consensus was performed using the CLC Genomics platform (Qiagen). In summary, the reads were mapped against a database containing representative sequences of all subtypes of influenza A virus. Next, the genes with the best scores of reads were selected to be mapped, and the consensus was recovered for further phylogenetic analysis. The obtained high-quality sequences were deposited in the EpiFlu database on the GISAID (Global Initiative on Sharing Avian Influenza Data) platform.

### Phylogeographic analysis.

Up to 100 genes displaying the closest identity (>95%) to the consensus sequence of each of the eight H11N2 gene segments were obtained by Basic Local Alignment Search Tool (BLAST) analysis against the complete GISAID/GenBank data set of H11N2 genes from avian hosts. To limit the data set dimensions but also keep its diversity, sequences identical in their nucleotide sequences, as determined by CD-HIT v.4.8.1 software (44), and in their continental regions of origin were removed from the final data set. The accession numbers of the retained sequences from the GISAID EpiFlu database can be found in the supplemental material. A global alignment of the retained data set for each of the segments (see Table S1 in the supplemental material) was then performed using MAFFT v.7.0 (45).

To inspect the temporal structure of the data sets, maximum likelihood (ML) phylogenetic trees were inferred using IQ-TREE v.2.1.3 (46) under the GTR+I+Γ4 nucleotide substitution model, as selected by the jModelTest program (47), and the subtree pruning & regrafting (SPR) branch-swapping algorithm for heuristic tree searching. The reliability of the obtained tree topology was estimated using the approximate likelihood ratio test (aLRT) (48) based on a Shimodaira-Hasegawa-like procedure. The association of divergence and sampling dates was then analyzed using Tempest v.1.5.3 (49).

The evolutionary rate, the time of the most recent common ancestor (*T*_MRCA_), and the phylogeographic dispersion pattern of the retrieved H11N2 samples were reconstructed by Bayesian inference using the BEAST v.1.10 software package (49–51), using BEAGLE (52) to improve the run time. Time-scaled phylogenies were inferred with the flexible Bayesian skyline (BSKL) coalescent model (53), using the GTR+I+Γ4 nucleotide substitution model and a relaxed uncorrelated log-normal molecular clock model (54). Migration events were modeled using a reversible discrete phylogeographic model (55) with a continous-time Markov chains (CTMC) rate reference prior (56) and five discrete locations, Asia, Antarctica, Europe, North America, and South America. Markov chain Monte Carlo (MCMC) chains were run for 100 × 10^6^ to 300 × 10^6^ generations, and the convergence and uncertainty of parameter estimates were assessed by calculating the effective sample size (ESS) and 95% highest probability density (HPD) values, respectively, after excluding the initial 10% of each run using Tracer v.1.7.1 (57). The convergence of parameters was considered when the ESS was ≥200. The maximum clade credibility (MCC) trees (MCCTs) were summarized using TreeAnnotator v.1.10.4 (51) and visualized using FigTree v.1.4.4 (https://github.com/rambaut/figtree/releases).

### Data availability.

The sequences are available at www.gisaid.org under the accession numbers EPI_ISL_2397324, EPI_ISL_2397326, EPI_ISL_2397327 and EPI_ISL_2397515.
