# Supplementary material for: Influenza A(H11N2) Virus Detection in Fecal Samples from Adélie (Pygoscelis adeliae) and Chinstrap (Pygoscelis antarcticus) Penguins, Penguin Island, Antarctica
Source: Microbiol Spectr. 2022 Sep 19;10(5):e01427-22. doi: 10.1128/spectrum.01427-22 (PMC9603087; doi:10.1128/spectrum.01427-22)
Supplement: Supplemental file 1 — Tables S1 to S3 and Fig. S1 and S2. Download spectrum.01427-22-s0002.pdf, PDF file, 0.8 MB [file spectrum.01427-22-s0002.pdf]

Supplementary Material

Supplementary Table 1: Datasets of H11N2 gene segments

Genes	Asia	Antarctica	Europe	North America	South America	Total
**PB2**	13	11	3	90	2	119
**PB1**	13	10	3	90	2	118
**PA**	13	13	3	30	2	121
**HA**	13	13	11	85	1	123
**NP**	13	12	12	90	2	129
**NA**	13	13	12	86	1	125
**MP**	13	14	12	88	2	129
**NS**	13	14	12	88	2	129

The table details the complete dataset used in the phylogeographic analysis of each of the eight H11N2's gene segments, detailing the absolute frequency of samples originating in different continents.

Supplementary Table 2: Estimated nucleotide substitution rated in H11N2 gene segments

Gene	Median	95% HPD
**PB2**	2.5E-3	2.0E-3 - 3.0E-3
**PB1**	2.3E-3	2.0E-3 - 2.8E-3
**PA**	1.8E-3	1.5E-3 - 2.1E-3
**HA**	4.3E-3	3.6E-3 - 5.2E-3
**NP**	2.3E-3	1.8E-3 - 2.8E-3
**NA**	3.5E-3	2.9E-3 - 4.3E-3
**MP**	2.0E-3	1.6E-3 - 2.5E-3
**NS**	2.5E-3	1.7E-3 - 3.6E-3

The table details the estimated nucleotide substitution rate estimated for each of H11N2*'s gene segments in an analysis done in Beast 1.10. Values were estimated as part of an uncorrelated log normal molecular clock.*

Supplementary Table 3: Spatiotemporal emergence of H11N2 Antarctic cluster.

**Gene**	**MRCA I**		**MRCA II**	
	**T_{MRCA}**	**95% HPD**	**Location**	**PSP**
**PB2**	2011	2010 - 2012	North America	1
**PB1**	2012	2012 - 2013	North America	0.35
**PA**	2012	2012 - 2013	North America	0.94
**HA**	2012	2012 - 2013	Europe	0.61
**NP**	2013	2012 - 2013	North America	0.99
**NA**	2012	2012 - 2013	North America	0.95
**MP**	2012	2011 - 2013	North America	0.99
**NS**	2010	2007 - 2012	North America	0.99

The table details, for each of H11N2's *gene segments, the time to the most recent common ancestor (T_{MRCA}) of the* H11N2 *Antarctic cluster inside this continent (MRCA I), as well as the most probable location, and the posterior state probability (PSP) of the first ancestor of the cluster outside the Antarctic continent (MRCA II). T_{MRCA} were inferred based on the median height of the Antarctic cluster in each run, and it is accompanied by its 95% highest posterior density (HPD) interval.*

Supplementary Figures

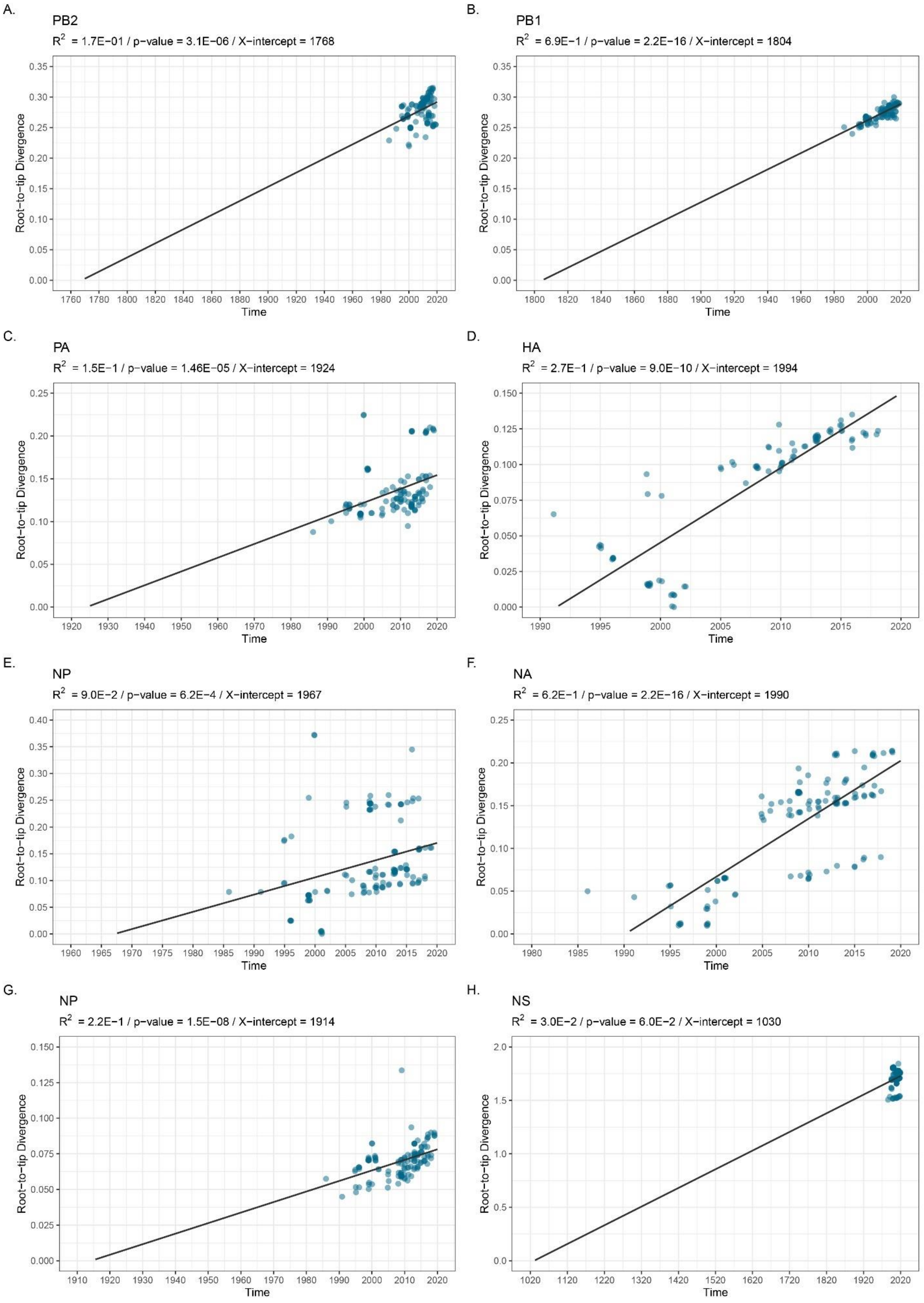
A.
PB2
R^2 = 1.7E−01 / p−value = 3.1E−06 / X−intercept = 1768
Root−to−tip Divergence
Time
B.
PB1
R^2 = 6.9E−1 / p−value = 2.2E−16 / X−intercept = 1804
Root−to−tip Divergence
Time
C.
PA
R^2 = 1.5E−1 / p−value = 1.46E−05 / X−intercept = 1924
Root−to−tip Divergence
Time
D.
HA
R^2 = 2.7E−1 / p−value = 9.0E−10 / X−intercept = 1994
Root−to−tip Divergence
Time
E.
NP
R^2 = 9.0E−2 / p−value = 6.2E−4 / X−intercept = 1967
Root−to−tip Divergence
Time
F.
NA
R^2 = 6.2E−1 / p−value = 2.2E−16 / X−intercept = 1990
Root−to−tip Divergence
Time
G.
NP
R^2 = 2.2E−1 / p−value = 1.5E−08 / X−intercept = 1914
Root−to−tip Divergence
Time
H.
NS
R^2 = 3.0E−2 / p−value = 6.0E−2 / X−intercept = 1030
Root−to−tip Divergence
Time

Supplementary Figure S1: **Analysis of the temporal structure of H11N2's gene segments datasets**. The graphs depict linear regressions of the root-to-tip divergence against sampling date observed in the sequences composing the H11N2 gene segments datasets: A) PB2, B) PB1, C) PA, D) HA, E) NP, F) NA, G) GA, and H) HG. The 'best-fitting' root for the phylogeny was found by maximizing the R^2 value of the root-to-tip regression. The X-intercept, the R^2 value, and the *p*-value are indicated in each graph.

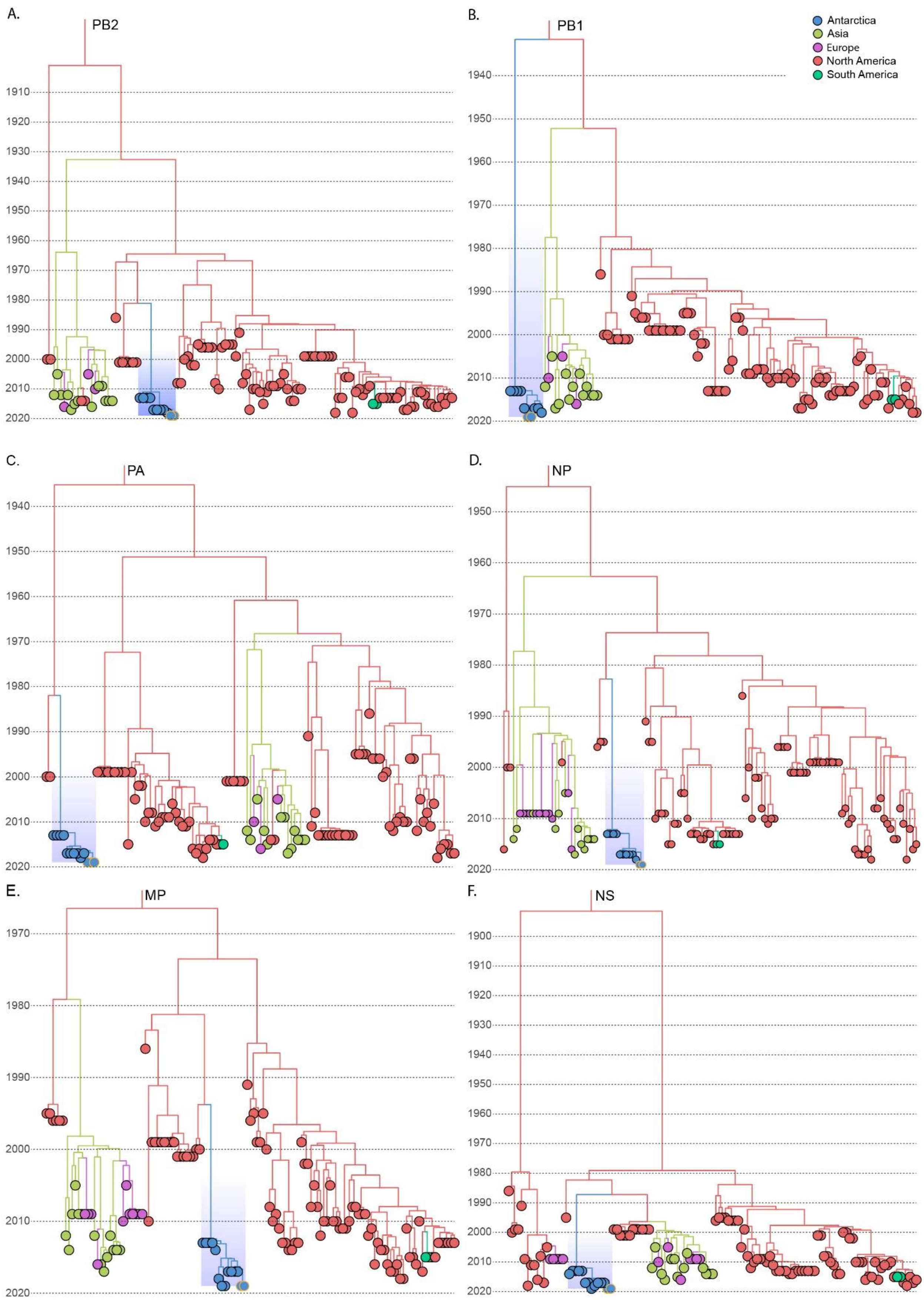

A.
PB2
B.
PB1
Antarctica
Asia
Europe
North America
South America
C.
PA
D.
NP
E.
MP
F.
NS

Supplementary Figure 2: Phylogeographic analysis of the PB2, PB1, PA, NP, MP and NS of the Influenza A H11N2 Antarctic samples Time-scaled Bayesian maximum clade credibility (MCC) trees of the PB1 (A, *n* = 119), PB2 (B, *n* = 118), PA (C, *n* = 121), NP (D, *n* = 129), MP (E, *n* = 129), and NS (F, *n* = 129) genes. The inhered location of tips and nodes in the trees are represented according to the color code present in the upper right corner of the figure. The Antarctic cluster is highlighted in light blue in all trees. Sequences from this study are highlighted by and orange outline.
